# Ferritin nanoparticles for improved self-renewal and differentiation of human neural stem cells

**DOI:** 10.1186/s40824-018-0117-y

**Published:** 2018-02-27

**Authors:** Jung Seung Lee, Kisuk Yang, Ann-Na Cho, Seung-Woo Cho

**Affiliations:** Department of Biotechnology, Yonsei University, 50 Yonsei-ro, Seodaemun-gu, Seoul, 03722 Republic of Korea

**Keywords:** Ferritin, Neural stem cell, Neurosphere, Self-renewal, Differentiation

## Abstract

**Background:**

Biomaterials that promote the self-renewal ability and differentiation capacity of neural stem cells (NSCs) are desirable for improving stem cell therapy to treat neurodegenerative diseases. Incorporation of micro- and nanoparticles into stem cell culture has gained great attention for the control of stem cell behaviors, including proliferation and differentiation.

**Method:**

In this study, ferritin, an iron-containing natural protein nanoparticle, was applied as a biomaterial to improve the self-renewal and differentiation of NSCs and neural progenitor cells (NPCs). Ferritin nanoparticles were added to NSC or NPC culture during cell growth, allowing for incorporation of ferritin nanoparticles during neurosphere formation.

**Results:**

Compared to neurospheres without ferritin treatment, neurospheres with ferritin nanoparticles showed significantly promoted self-renewal and cell-cell interactions. When spontaneous differentiation of neurospheres was induced during culture without mitogenic factors, neuronal differentiation was enhanced in the ferritin-treated neurospheres.

**Conclusions:**

In conclusion, we found that natural nanoparticles can be used to improve the self-renewal ability and differentiation potential of NSCs and NPCs, which can be applied in neural tissue engineering and cell therapy for neurodegenerative diseases.

**Electronic supplementary material:**

The online version of this article (10.1186/s40824-018-0117-y) contains supplementary material, which is available to authorized users.

## Background

With a worldwide increase in expected lifespan, the number of patients suffering from neurodegenerative disorders (e.g. Alzheimer’s disease, Parkinson’s disease, and Huntington’s disease) due to the progressive death of neurons has consistently increased [1, 2]. Neural stem cells (NSCs), which have self-renewal ability and can differentiate into neurons, oligodendrocytes, and astrocytes have been considered as a primary cell source for cell therapy to regenerate neural tissue and cure neurological diseases [3, 4]. To potentiate the therapeutic efficacy and regenerative capacity of NSCs, controlling the self-renewal and differentiation of NSCs is important. Therefore, several studies have suggested biomaterial-based strategies for promoting the self-renewal and differentiation of NSCs using functional hydrogels, biomimetic scaffolds, patterned substrates, and particulates [5–9].

Previously, incorporation of micro- and nanoparticles into stem cell culture was shown to be an efficient method for controlling various cellular behaviors of stem cells. For example, polymeric nanoparticles increased the rate of cellular aggregation and enhanced phenotypic characteristics including lineage-specific gene expression and functionalities [10, 11]. The enhanced gap junction formation of stem cells by iron oxide nanoparticles was previously reported to improve therapeutic efficacy of stem cells for ischemia treatment [12]. Sustained growth factor delivery from incorporated microparticles into embryonic stem cells (ESCs) enhanced vascular differentiation of embryoid bodies (EBs) [13] and the addition of conductive particles significantly enhanced neuronal differentiation of NSCs [9] as well as cardiac differentiation of ESCs [11]. In NSC culture, the stage of neurosphere formation for NSC expansion is a critical step that can determine the self-renewal ability and differentiation potential of NSCs [14, 15]. However, the heterogeneous size distribution and lack of neurogenic capacity of neurospheres generated through conventional floating culture methods are critical limitations that must be overcome to produce therapeutic NSCs exhibiting increased proliferative ability and neuronal differentiation capacity [14]. Because the addition of engineered particles during stem cell spheroid formation largely affects the self-renewal and differentiation capacity of stem cells [9–11, 13], the particle types of biomaterials should be considered to improve cell therapeutics derived from NSC neurospheres.

In this study, we added a naturally derived nanoparticle, ferritin, during the generation of NSC neurospheres and investigated its effects on the self-renewal ability and differentiation of NSCs. Ferritin, an iron-containing protein nanoparticle that controls iron concentration in vivo, has recently been utilized as a natural biomaterial for various biomedical applications such as drug delivery [16], gene delivery [17], in vivo imaging [18], and cancer treatment [19, 20] because of its intrinsic biochemical and structural characteristics. Herein, ferritin nanoparticles were incorporated during neurosphere formation of two types of neuronal lineage stem/progenitor cells: human fetal NSCs (hfNSCs) and human induced pluripotent stem cell (hiPSC)-derived neural progenitor cells (NPCs) (hiPSC-NPCs). The optimal concentration of ferritin nanoparticles was demonstrated to generate uniformly sized neurospheres and enhance NSC self-renewal with minimal cytotoxicity. Importantly, ferritin nanoparticle treatment significantly enhanced the neuronal differentiation capacity of the NSCs and NPCs, indicating that the therapeutic efficacy of stem cells can be potentiated by nanoparticle-based engineering of neurosphere formation.

## Methods

### Cell culture

hfNSCs isolated from the telencephalon at 13 weeks of gestation were kindly provided by Prof. Kook In Park, Yonsei University College of Medicine and expanded as previously reported [6]. Briefly, hfNSCs were seeded onto a petri-dish (Corning, Inc., Corning, NY, USA) at a density of 6.0 × 10^5^ cells/mL and expanded to form neurospheres in Dulbecco’s Modified Eagle’s Medium: Nutrient Mixture F12 (DMEM/F12, Gibco, Grand Island, NY, USA) supplemented with N-2 supplement (Gibco), basic fibroblast growth factor (bFGF, 20 ng/mL, Sigma, St. Louis, MO, USA), and leukemia inhibitory factor (LIF, 10 ng/mL, Sigma). For spontaneous differentiation of hfNSCs, the cells were cultured in expansion medium deprived of mitogenic factors (bFGF and LIF).

hiPSCs (cell line: WT3) were kindly provided by Prof. Dong-Wook Kim, Yonsei University College of Medicine. Experiments were conducted with the approval of the Institutional Review Board of Yonsei University (1040927–201,510-BR-229-01E). hiPSCs were maintained on feeder cell layers of STO fibroblasts (American Type Culture Collection, Manassas, VA, USA) as described in a previous study [8]. The cells were detached and cultured on a non-adherent petri-dish to form EBs for subsequent neuronal differentiation in DMEM/F12 supplemented with knockout-serum replacement (20%, Invitrogen, Carlsbad, CA, USA), penicillin/streptomycin (1%, Invitrogen), nonessential amino acids (Invitrogen), β-mercaptoethanol (0.1 mM, Sigma), dorsomorphin (5 μM, Sigma), and SB431542 (5 μM, Sigma). After 4 days of culture, formed EBs were attached to a Matrigel (BD Biosciences, San Jose, CA, USA)-coated culture dish and further differentiated for 4–5 days in neuronal induction medium composed of DMEM/F12 supplemented with N-2 supplement (Invitrogen) and nonessential amino acids (Invitrogen) [21]. hiPSC-NPCs were acquired by mechanical collection of cell clusters from the center of the attached EBs and then dissociated into single cells by accutase (Invitrogen) [22]. Single hiPSC-NPCs at a density of 6.0 × 10^5^ cells/mL were aggregated to form neurospheres in microwells (SpheroFilm™, 200 μm inner diameter, inCYTO, Chonan, Korea) in neuronal induction medium containing Y27632 (10 μM, Sigma). For differentiation of hiPSC-NPCs, the cells were cultured in neuronal induction medium.

Primary mouse neurons were isolated from hippocampus of ICR mouse embryos (E14, Orient Bio, Sungnam, Korea), as previously described [23]. The isolated primary neurons were cultured on the poly-_L_-lysine- (PLL, 20 μg/mL, Sigma) and laminin (2.5 μg/mL, Sigma)-coated substrates using neurobasal medium (Gibco) supplemented with 1× B27 (Gibco), 1× GlutaMAX (Gibco), and penicillin/streptomycin (1%, Gibco). All cells were cultured at 37 °C in humidified air with 5% CO_2_.

### Incorporation of ferritin nanoparticles

Ferritin from equine spleen was purchased from Sigma. For hfNSCs, ferritin nanoparticles were added to the cells by providing expansion medium containing ferritin at different concentrations (0.02, 0.1, and 0.3 mg/mL), and the cells were allowed to form neurospheres for 6 days. Because ferritin nanoparticles can be internalized by receptor-mediated endocytosis [24], to minimize change in the concentration of ferritin nanoparticles in culture medium during neurosphere formation, ferritin nanoparticles were continuously supplemented to cells by providing ferritin-containing medium at the same ferritin concentration whenever the culture medium was replaced every 2 days. For hiPSC-NPCs, ferritin was treated during the first 2 days of single-cell aggregation in microwells. For differentiation induction, neurospheres of ferritin-treated hfNSCs and hiPSC-NPCs were transferred onto PLL- (20 μg/mL, Sigma) and fibronectin (10 μg/mL, R&D Systems, Minneapolis, MN, USA)-coated culture substrates and maintained under differentiation medium conditions for 4 days.

### Size distribution measurement

To analyze the size distribution of neurospheres, neurospheres formed after ferritin treatment (6 days for hfNSCs and 2 days for hiPSC-NPCs) were collected and their diameters were measured using Image J software (National Institute of Health, Bethesda, MD, USA).

### Viability and proliferation test

Viable cells in the generated neurospheres after ferritin treatment were stained with Live/Dead viability/cytotoxicity kit (Invitrogen) and imaged using a fluorescent microscope (IX71, Olympus, Tokyo, Japan). Metabolic activity of ferritin-treated cells was measured in a 3-(4,5-dimethylthiazol-2-yl)-2,5-diphenyltetrazolium bromide (MTT, Sigma) assay. After aggregation of cells treated with ferritin nanoparticles (6 days for hfNSCs and 2 days for hiPSC-NPCs), the neurospheres were collected and treated with MTT solution (5 mg/mL) for 4 h. Dimethyl sulfoxide (Sigma) was used to solubilize the MTT crystals and the absorbance at 560 nm was measured using a microplate reader (Tecan, Männedorf, Switzerland). For the proliferation test, MTT assay was performed 2 and 5 days of the culture with ferritin treatment. The absorbance of each group at day 5 was normalized to the value at day 2.

### Quantitative real-time polymerase chain reaction (qPCR)

To extract total RNA from cells, an RNeasy Mini Kit (Qiagen, Hilden, Germany) was used. cDNA synthesis was carried out using the PrimeScript II first-strand cDNA Synthesis kit (Takara, Shiga, Japan). A StepOnePlus Real-Time PCR System (Applied Biosystems, Foster City, CA, USA) and TaqMan Fast Universal PCR Master Mix (Applied Biosystems) were used for qPCR analysis. The level of gene expression in each sample was examined in TaqMan Gene Expression Assays (Applied Biosystems) for human Nestin (Hs04187831_g1), human OCT4 (Hs00742896_s1), human Nanog (Hs02387400_g1), human cadherin 1 (CDH1, Hs01023894_m1), human cadherin 2 (CDH2, Hs00983056_m1), human neuronal class III β-tubulin (Tuj1, Hs00801390_s1), human microtubule associated protein 2 (MAP2, Hs00258900_m1), human oligodendrocyte transcription factor 2 (Olig2, Hs00300164_s1), and human glial fibrillary acidic protein (GFAP, Hs00909238_g1). Relative expression was evaluated by the comparative *C*_t_ method and the expression value for each marker was normalized to that of the endogenous reference transcript, human glyceraldehyde-3-phosphate dehydrogenase (Hs02758991_g1).

### Immunostaining

For immunostaining, cells were fixed with 10% (v/v) formalin (Sigma) for 10 min, permeabilized with 0.1% (v/v) Triton X-100 (Wako, Osaka, Japan) for 10 min, and treated with 5% (w/v) bovine serum albumin (Sigma) for 30 min to block nonspecific binding of antibodies. The samples were incubated with primary antibodies overnight at 4 °C and then with secondary antibodies for 1 h at room temperature. The following primary antibodies were used: anti-Nestin (1:100, Millipore, Billerica, MA, USA), anti-N-cadherin (1:200, Santa Cruz Biotechnology, Dallas, TX, USA), anti-Tuj1 (1:200, Cell Signaling Technology, Danvers, MA, USA), anti-NeuN (1:400, Millipore), and anti-GFAP (Cell Signaling Technology). The following secondary antibodies were used: Alexa-Fluor 488 goat anti-mouse IgG antibody (1:200, Invitrogen) and Alexa-Fluor 594 donkey anti-rabbit IgG antibody (1:200, Invitrogen). The nuclei were counterstained using 4′-6-diamidino-2-phenylindole (DAPI, Sigma). Stained cells were imaged using a confocal microscope (LSM 700, Carl Zeiss, Jena, Germany).

### Statistical analysis

All quantitative data are represented as the mean ± standard deviation and analyzed with GraphPad Prism software (GraphPad Software, Inc., La Jolla, CA, USA). Statistical significance was evaluated using an unpaired Student’s *t* test and *p*-values under 0.01 or 0.05 were considered statistically significant.

## Results

### Effects of ferritin nanoparticles in neurosphere formation

**Fig. 1:**
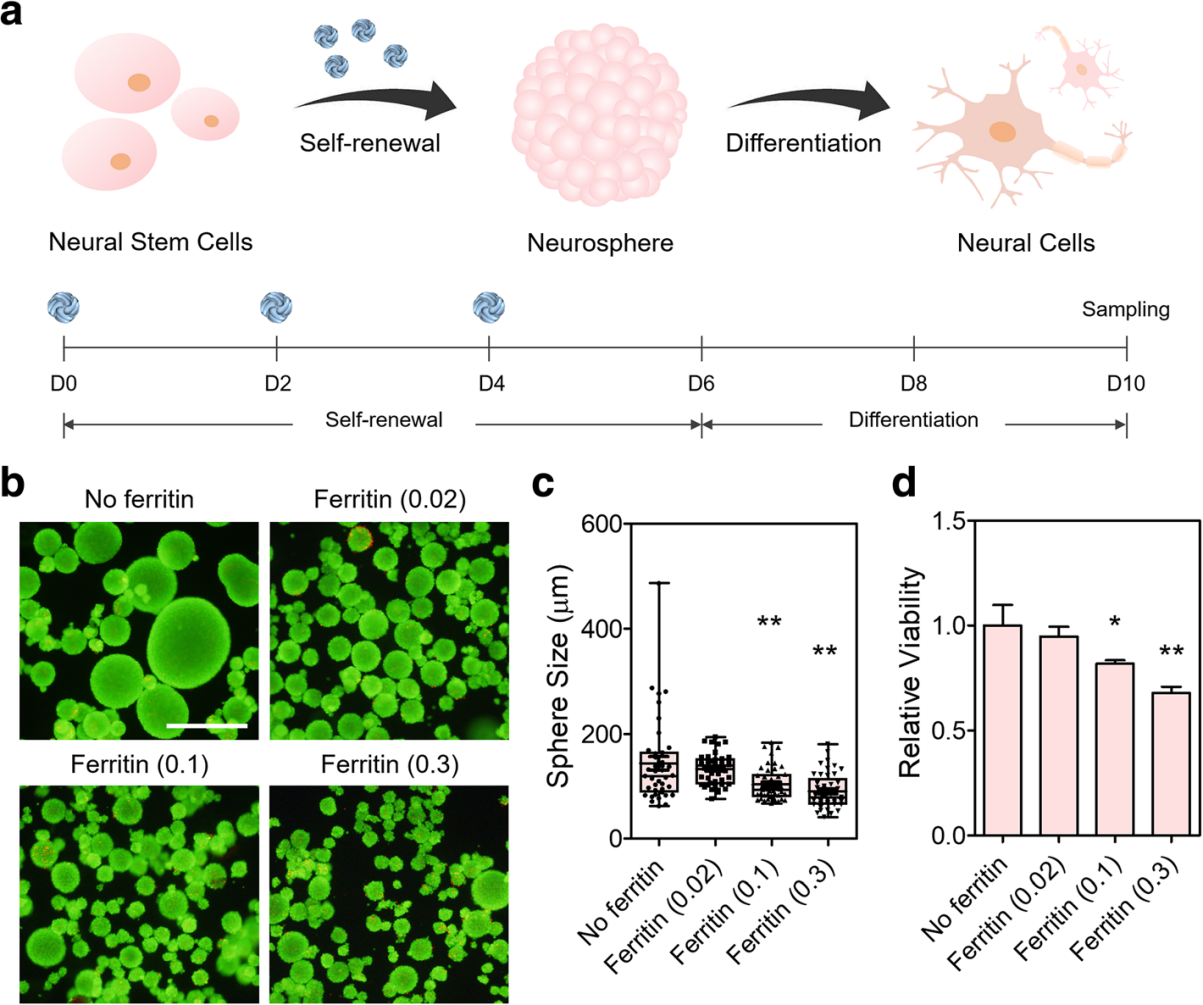
Effect of ferritin incorporation on neurosphere formation. **a** Schematic illustration and timeline of the experiments. Ferritin nanoparticles were incorporated into neurospheres by addition to the culture media (0.02, 0.1, and 0.3 mg/mL) every 2 days during culture to expand hfNSCs. Subsequently, neurospheres were plated onto cell culture plates coated with fibronectin and allowed to differentiate spontaneously for 4 days. Medium was exchanged every 2 days. **b** Neurospheres of hfNSCs cultured with or without ferritins were stained with calcein-AM (for live cells; green) and ethidium homodimer-1 (for dead cells; red) after 6 days of culture for self-renewal and expansion. Scale bar = 500 μm. **c** Average size of generated hfNSC neurospheres in each group after 6 days of culture (*n* = 40–45, **p < 0.01 versus No ferritin group). **d** Relative viability of hfNSCs in each group after 6 days of culture under self-renewal conditions, evaluated by MTT assay (*n* = 4, *p < 0.05 and **p < 0.01 versus No ferritin group)

Established methods for incorporating micro- and nanoparticles during stem cell culture typically require complicated synthetic fabrication processes [9, 11, 13] and may require improvements in biocompatibility. Thus, naturally derived particulates may provide an efficient platform for stem cell engineering. Among them, ferritin, a natural protein nanoparticle 8–12 nm in size, which is abundant in the serum and plays important roles in precisely regulating cellular iron concentrations critical for neuronal development [25], is candidate biomaterial for engineering NSC or NPC culture. To examine the effects of ferritin nanoparticles on the neurosphere formation of hfNSCs, cells were cultured in an expansion medium supplemented with mitogenic factors (bFGF and LIF) and ferritin for 6 days, and then the formed neurospheres were transferred onto cell culture plates for subsequent spontaneous differentiation (Fig. 1a). When ferritin nanoparticles were added at various concentrations (0.02, 0.1, and 0.3 mg/mL) during the culture of hfNSCs in the form of neurospheres, most cells in the neurospheres were viable (Fig. 1b) and the size of neurospheres became more homogenous regardless of the ferritin concentration compared to those cultured without ferritin (Fig. 1b, c). Because the size of stem cell spheroids is known to largely affect the self-renewal ability and differentiation capacity of stem cells [14, 15], generating neurospheres with a uniform size distribution is important for preparing a homogeneous cell population that exhibits enhanced therapeutic efficacy. Interestingly, the average size of the generated neurospheres gradually decreased as ferritin concentration increased (Fig. 1c, no ferritin; 143.5 ± 82.1 μm, 0.02 mg/mL ferritin; 132.9 ± 29.4 μm, 0.1 mg/mL ferritin; 103.3 ± 30.8 μm, and 0.3 mg/mL ferritin; 89.8 ± 33.6 μm), likely because of the formation of more compact neurospheres induced by increased concentrations of ferritin nanoparticles.

Next, the cytocompatibility of ferritin nanoparticles with hfNSCs was examined by the MTT assay after 2- and 6-day culture with ferritin addition. Ferritin treatment for 2 days did not induce cytotoxicity at 0.02 mg/mL, but as the concentration of ferritin nanoparticles increased up to 0.3 mg/mL, the viability of ferritin-treated hfNSCs gradually decreased, indicating the cytotoxic effect by ferritin nanoparticles of higher concentrations (Additional file 1: Figure S1). The relative viability of hfNSCs to the no ferritin group at day 6 also decreased as ferritin concentration increased to 0.1 and 0.3 mg/mL (Fig. 1d). Particularly, the 0.3 mg/mL ferritin group showed significantly lower relative viability (68.1 ± 3.0%) than the no ferritin group, indicating that higher concentrations of ferritin were cytotoxic to hfNSCs (Fig. 1d). Therefore, the 0.3 mg/mL ferritin group was excluded from subsequent experiments. The ferritin-treated hfNSCs proliferated over culture time, but when compared with non-treated cells, they exhibited less proliferative ability 5 days of the culture, even in 0.02 mg/mL group that did not show cytotoxicity (Additional file 1: Figure S2). This result may indicate that the proliferative ability of hfNSCs might be slightly impaired by ferritin treatment. Since the proliferation rate of hfNSCs was not increased by ferritin treatment, there was no significantly detectable difference in the length of time for neurosphere formation.

### Enhanced self-renewal of hfNSCs by ferritin nanoparticle incorporation

**Fig. 2:**
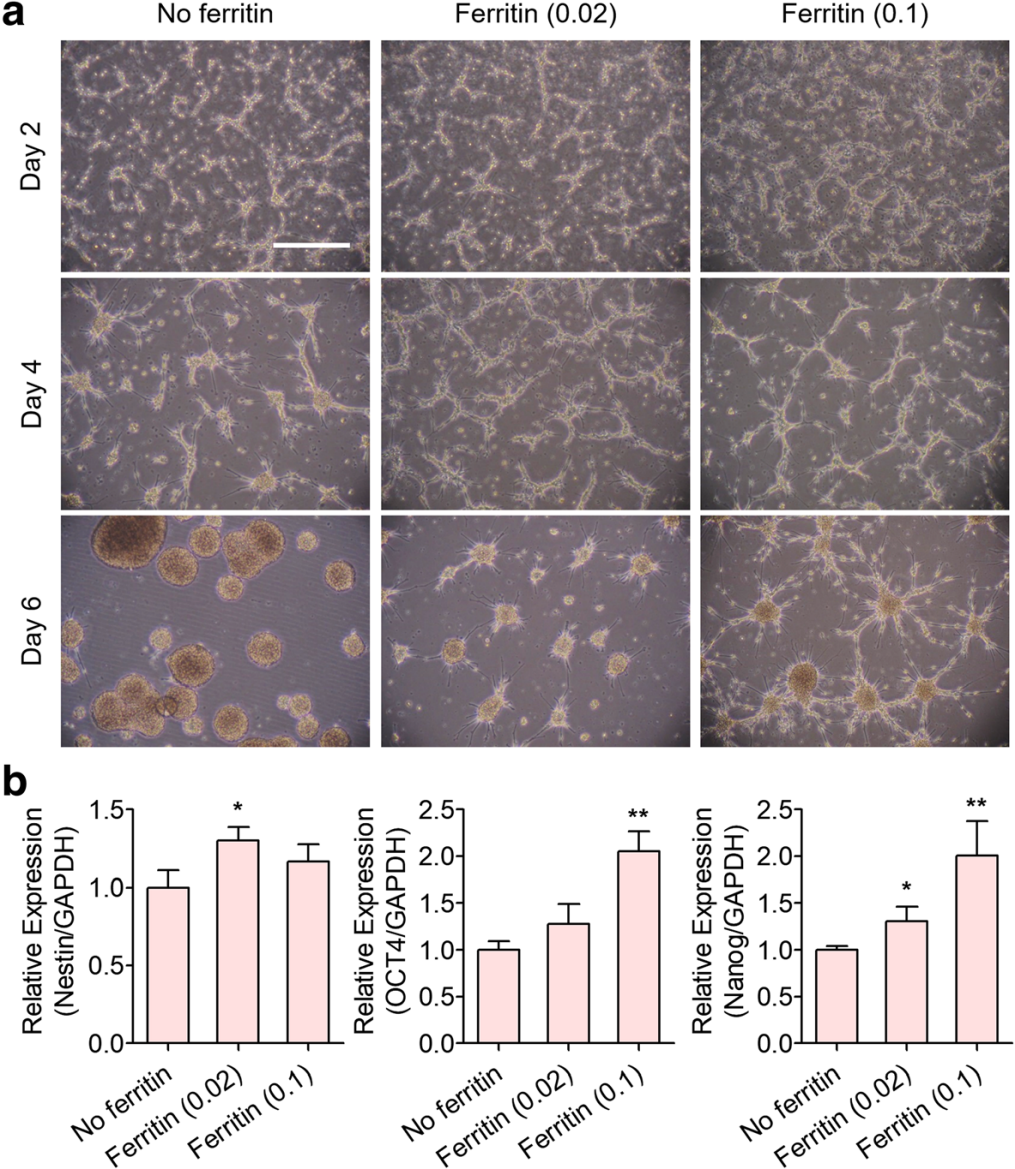
Enhancement of self-renewal of hfNSCs by ferritin incorporation. **a** Microscopic observation of hfNSCs during culture for self-renewal and expansion with or without ferritin treatment. Scale bar = 200 μm. **b** qPCR analysis for evaluating gene expression of neuronal progenitor and stemness markers (Nestin, OCT4, and Nanog) in the generated neurospheres after 6 days of culture (*n* = 3, *p < 0.05 and **p < 0.01 versus No ferritin group)

**Fig. 3:**
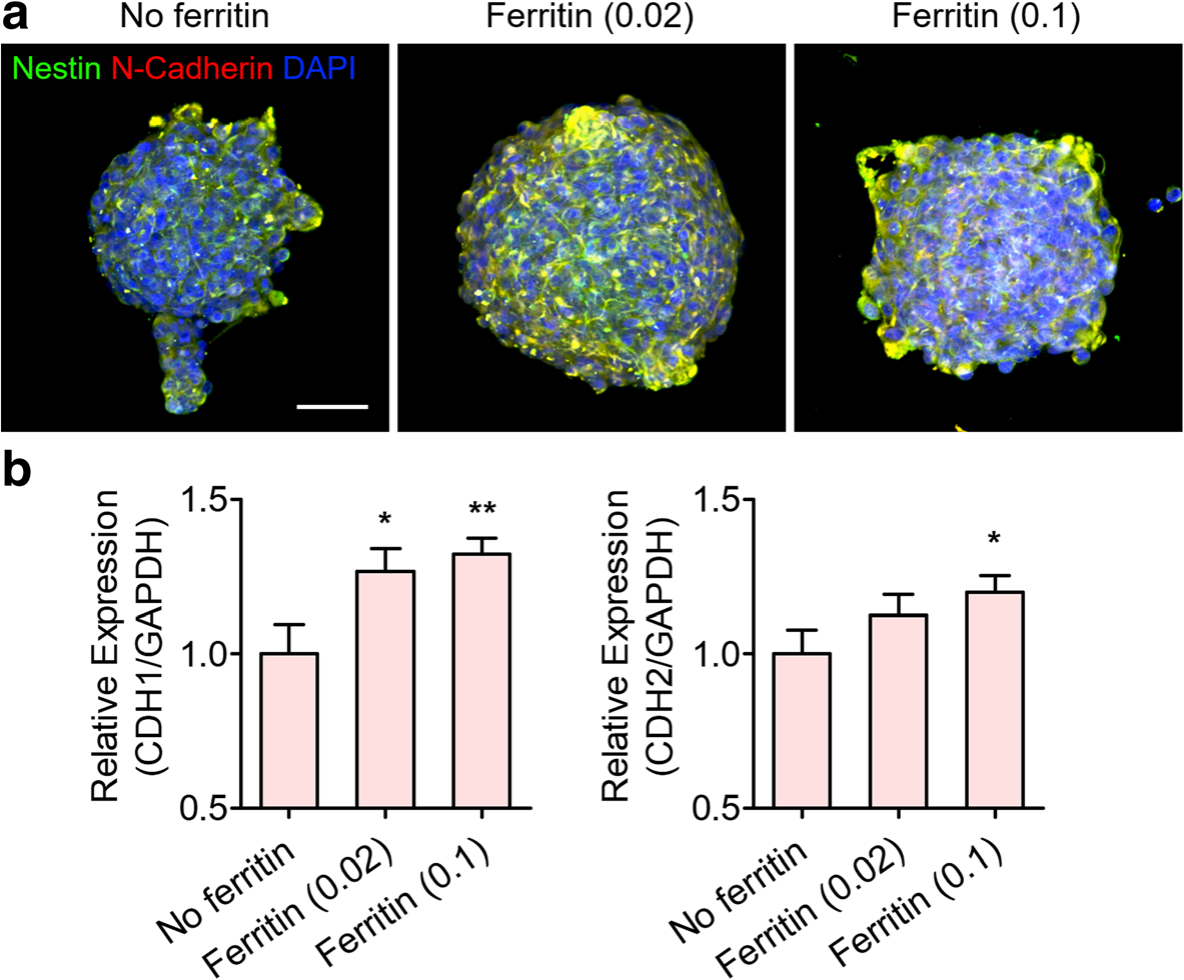
Increased cell-cell interactions in hfNSC neurospheres by ferritin nanoparticle incorporation after 6 days of culture under expansion media conditions. **a** Immunofluorescence staining for Nestin (green) and N-cadherin (red) of hfNSC neurospheres. Cell nuclei were counterstained with DAPI. Scale bar = 50 μm. **b** qPCR analysis for evaluating gene expression of adhesion molecules related to cell-cell interactions including E-cadherin (CDH1) and N-cadherin (CDH2) (*n* = 3, *p < 0.05 and **p < 0.01 versus No ferritin group)

We investigated whether ferritin treatment promotes self-renewal of hfNSCs. There was no detectable difference in neurosphere formation among groups at the early stage of expansion (~ 4 days) under self-renewal conditions with mitogenic factors. However, the formed neurospheres in the no ferritin group began to merge after 4 days of culture, resulting in neurospheres with a heterogeneous size distribution (Figs. 1c and 2a). As described above, ferritin incorporation during neurosphere formation induced more homogeneous formation of hfNSC neurospheres (Fig. 2a). To evaluate the effect of ferritin incorporation on the self-renewal of hfNSCs, gene expression levels of stemness and progenitor markers were compared by qPCR analysis between the no ferritin and ferritin-treated groups (Fig. 2b). After 6 days of culture under self-renewal conditions, gene expression of all tested markers, including Nestin, OCT4, and Nanog, was increased in the ferritin-treated groups compared to in the no ferritin group (Fig. 2b). Nestin expression in neurospheres was highest in the 0.02 mg/mL ferritin treatment group. OCT4 and Nanog expression levels in neurospheres were upregulated in a ferritin dose-dependent manner, demonstrating that 0.1 mg/mL of ferritin treatment led to the highest expression of the two markers (Fig. 2b). Increased expression of the neural progenitor marker Nestin in the 0.02 and 0.1 mg/mL ferritin groups was further confirmed by immunostaining for Nestin in neurospheres (Fig. 3a). These results demonstrate that ferritin incorporation enhanced the self-renewal and stemness of hfNSCs.

Importantly, ferritin nanoparticle incorporation into neurospheres promoted cell-cell interactions, which may explain the enhanced self-renewal and stemness of hfNSCs in neurospheres treated with ferritin. N-Cadherin, also known as cadherin-2 (CDH2), which is involved in adherent junction formation [26], was highly expressed in ferritin-treated neurospheres (Fig. 3a). In addition to N-cadherin, the expression of E-cadherin (cadherin-1; CDH1), another representative cell-cell adhesion molecule, was significantly upregulated in ferritin-treated groups, as confirmed by qPCR analysis (Fig. 3b). Cell-cell interactions are crucial for maintaining and enhancing the self-renewal of stem cells [27, 28]. Thus, the improved self-renewal capacity of hfNSCs in ferritin-treated neurospheres may be because of cell-cell interactions facilitated by incorporated ferritin nanoparticles. Particularly, a ferritin concentration of 0.1 mg/mL resulted in the generation of homogeneous and compact stem cell aggregates with increased stemness and self-renewal abilities.

### Enhanced neuronal differentiation of hfNSC neurospheres by ferritin nanoparticle incorporation

**Fig. 4:**
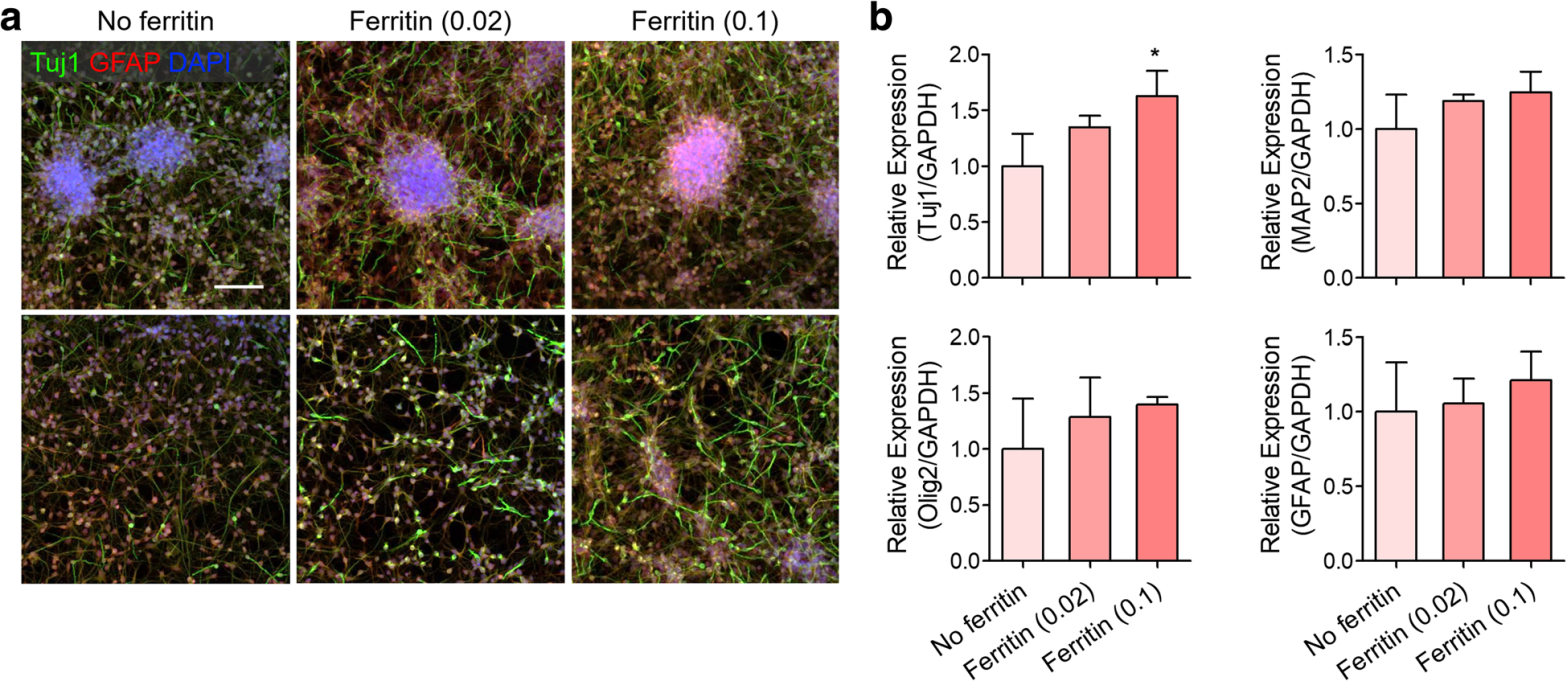
Promoted differentiation capacity of hfNSC neurospheres by ferritin incorporation. **a** Immunofluorescence staining for Tuj1 (green) and GFAP (red) of the differentiated hfNSCs near (upper row) and apart from (lower row) the attached neurospheres after 4 days under spontaneous differentiation culture conditions. Cell nuclei were counterstained with DAPI. Scale bar = 200 μm. **b** qPCR analysis for evaluating gene expression of Tuj1, MAP2, Olig2, and GFAP in differentiated hfNSC neurospheres (*n* = 3, *p < 0.05 versus No ferritin group)

Next, we examined the effects of ferritin nanoparticles on hfNSC differentiation. To initiate spontaneous differentiation of hfNSCs, generated neurospheres with or without ferritin treatment were transferred onto fibronectin-coated cell culture plates without mitogenic factors. Culture for spontaneous differentiation was conducted without additional ferritin treatment to solely evaluate the effects of ferritin nanoparticles incorporated into the neurospheres. After transferring the neurospheres onto the culture plates, hfNSCs migrated from the attached neurospheres and showed extended cellular morphology (Fig. 4a). Immunostaining of differentiated hfNSCs for neuronal class III β-tubulin (Tuj1) and glial fibrillary acidic protein (GFAP) was conducted to evaluate lineage-specific differentiation after 4 days of spontaneous differentiation (Fig. 4a). Tuj1 and GFAP are representative markers for evaluating neuronal and glial differentiation, respectively [6]. Differentiated hfNSCs from the attached neurospheres showed upregulated Tuj1 expression and enhanced neurite extension in ferritin-treated groups, particularly in the 0.1 mg/mL ferritin group (Fig. 4a), which is similar to those of primary neurons (Additional file 1: Figure S3), indicating that ferritin nanoparticle incorporation into neurospheres indirectly facilitated neuronal differentiation of hfNSCs probably due to the promoted stemness and self-renewal of hfNSCs by ferritin treatment. These results were also confirmed by qPCR analysis, which revealed increased gene expression of Tuj1 in 0.02 and 0.1 mg/mL ferritin-treated groups (Fig. 4b). Although the differences were not significant, gene expression of other neuronal markers (microtubule-associated protein 2, MAP2), oligodendrocyte lineage marker (oligodendrocyte transcription factor 2, Olig2), and GFAP, was also slightly increased in hfNSCs differentiated from ferritin nanoparticle-incorporated neurospheres (Fig. 4b).

### Promotion of hiPSC-NPC differentiation by ferritin nanoparticle incorporation

**Fig. 5:**
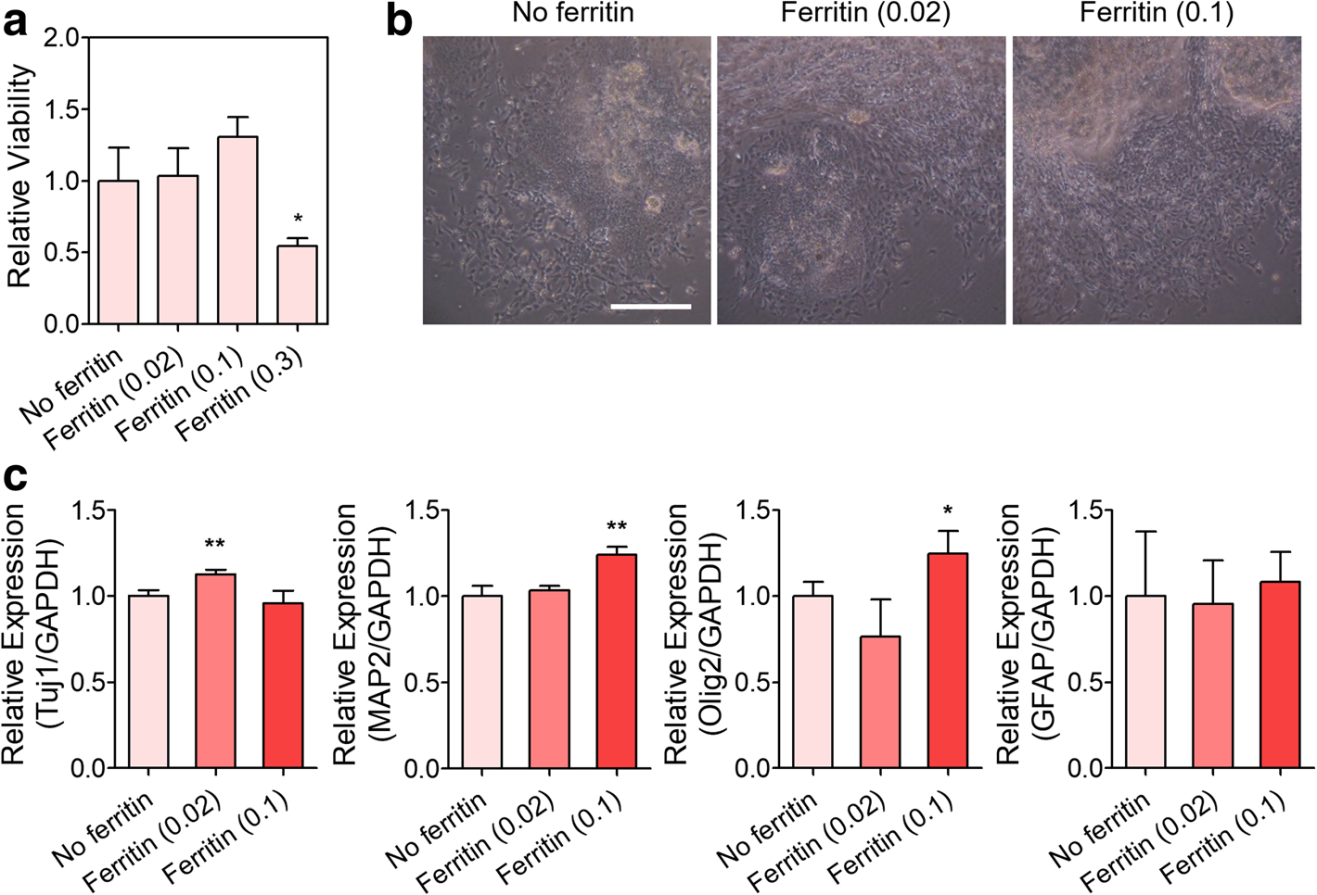
Effects of ferritin nanoparticle incorporation on hiPSC-NPC neurospheres. **a** Relative viability of hiPSC-NPCs after 2 days of ferritin treatment, as examined by MTT assay (*n* = 3, *p < 0.05 versus No ferritin group). **b** Microscopic observation of differentiated hiPSC-NPCs from attached neurospheres after 4 days under differentiation conditions. Scale bar = 200 μm. **c** qPCR analysis for evaluating gene expression of Tuj1, MAP2, Olig2, and GFAP (*n* = 3, *p < 0.05 and **p < 0.01 versus No ferritin group)

Finally, we examined whether treatment of ferritin nanoparticles affected the differentiation of hiPSC-derived NPCs, another promising cell source for treating neurodegenerative diseases [29]. hiPSC-derived EBs were further differentiated into neural rosettes, a type of NPC, and the collected NPCs were dissociated into single cells and aggregated to form neurospheres using a microwell device. Various concentrations of ferritin (0.02, 0.1, and 0.3 mg/mL) were added to the cells during the formation of hiPSC-NPC neurospheres. MTT assay demonstrated that only 0.3 mg/mL ferritin significantly decreased cell viability, while lower concentrations of ferritin (0.02 and 0.1 mg/mL) did not induce cytotoxicity to hiPSC-NPCs (Fig. 5a). Accordingly, similar to the results for hfNSCs, 0.3 mg/mL ferritin was excluded from subsequent differentiation experiments because of cytotoxicity. hiPSC-NPC neurospheres cultured with or without ferritin in the microwells were collected and transferred onto a fibronectin-coated culture plate, and then differentiation was induced without additional ferritin treatment. Differentiated cells in all groups actively migrated and spread out from the neurospheres (Fig. 5b). After 4 days of differentiation culture, qPCR analysis to examine the gene expression of differentiation markers confirmed that the neuronal markers Tuj1 and MAP2 was upregulated in the ferritin-incorporated neurosphere groups (Fig. 5c). Interestingly, Tuj1 was highly expressed in the presence of 0.02 mg/mL ferritin, while the expression of MAP2 was significantly enhanced in the 0.1 mg/mL ferritin group. In addition, higher concentrations of ferritin nanoparticles promoted Olig2 expression, but there was no significant difference in GFAP expression between the ferritin treatment and no ferritin groups. These results support that ferritin incorporation during neurosphere formation enhances the differentiation capacity of NPCs, particularly differentiation into the neuronal lineage.

## Discussion

Based on our results, ferritin nanoparticles mediate the formation of compact neurospheres by improving cell-cell interactions and significantly promote the self-renewal capacity of hfNSCs, resulting in enhanced neuronal differentiation under differentiation-inducing conditions. However, we only evaluated short-term differentiation of hfNSCs (~ 4 days), and thus the long-term effects of incorporated ferritin nanoparticles on neuronal differentiation require further analysis. In this study, it seems that incorporation of ferritin nanoparticles into neurospheres indirectly promotes stem cell neurogenesis via enhancement of self-renewal ability and stemness. Therefore, direct effects of ferritin nanoparticles on neuronal differentiation of stem cells should also be examined in future study.

Along with the role of ferritin nanoparticles in mediating cell-cell adhesion and enhancing cell-cell interactions in neurospheres, changing the iron concentration by ferritin as a biochemical factor may also alter the differentiation capacity of NSCs and NPCs. Iron concentration in the brain, which is typically regulated by iron-regulating proteins (e.g. ferritin and transferrin), is known to be crucial in central nervous system metabolism, myelination, and brain development [30–32]. Although high levels of iron accumulation can induce trauma and neuronal disease via oxidative cellular damage [33], iron is an essential metallic cofactor for the synthesis of enzymes and neurotransmitters for normal neuronal development [34]. Specifically, the high concentrations of iron, ferritin, and transferrin at birth indicate that a sufficient iron supply is closely related to normal neurological development [25]. Indeed, Lu et al. demonstrated that elevated iron concentrations dramatically accelerated neuronal differentiation of ESCs through a transferrin-mediated mechanism [35]. Therefore, increasing iron concentration by incorporating iron-containing ferritin nanoparticles may also contribute to enhanced neuronal differentiation of NSCs and NPCs, but the exact mechanism requires further evaluation.

## Conclusion

In this study, we developed an engineering approach for enhancing the self-renewal and differentiation capacity of NSCs and NPCs via simple modification using iron-containing natural ferritin nanoparticles. We identified the optimal concentration of ferritin treatment that induces homogeneous formation of neurospheres with minimal cytotoxicity. Importantly, incorporation of ferritin nanoparticles into neurospheres enhanced cell-cell interactions and self-renewal ability, ultimately leading to a greater neuronal differentiation capacity of NSCs and NPCs, although the exact mechanism requires further analysis. In conclusion, ferritin-mediated enhancement of the self-renewal and neuronal differentiation of NSCs and NPCs suggests the potential utility of using ferritin nanoparticles to improve NSC therapy and neural tissue engineering.

## Additional file

Additional file 1: **Figure S1.** The relative viability of hfNSCs in each group after 2 days of culture under self-renewal conditions, which was evaluated by MTT assay (*n* = 3, *p < 0.05 and **p < 0.01 versus No ferritin group). The viability of each group was normalized to that of the No ferritin group. **Figure S2.** The relative proliferation of hfNSCs in each group after 2 and 5 days of culture under self-renewal conditions, which was evaluated by MTT assay (n = 3, **p < 0.01 versus No ferritin group). The proliferation of each group at day 5 was normalized to that of each corresponding group at day 2. **Figure S3.** Immunofluorescence staining of primary hippocampal neurons for Tuj1 (green) and NeuN (red). Cell nuclei were counterstained with DAPI. Scale bar = 200 μm. (DOCX 1587 kb)

## Abbreviations

bFGF: Basic fibroblast growth factor
CDH 1: Cadherin 1
CDH 2: Cadherin 2
DAPI: 4′-6-diamidino-2-phenylindole
DMEM/F12: Dulbecco’s Modified Eagle’s Medium: Nutrient Mixture F12
EBs: Embryoid bodies
ESCs: Embryonic stem cells
GFAP: Glial fibrillary acidic protein
hfNSCs: Human fetal neural stem cells
hiPSC-NPCs: Human induced pluripotent stem cell-derived neural progenitor cells
hiPSCs: Human induced pluripotent stem cells
LIF: Leukemia inhibitory factor
MAP2: Microtubule associated protein 2
MTT: 3-(4,5-dimethylthiazol-2-yl)-2,5-diphenyltetrazolium bromide
NPCs: Neural progenitor cells
NSCs: Neural stem cells;
Olig2: Oligodendrocyte transcription factor 2
qPCR: Quantitative real-time polymerase chain reaction
Tuj1: Neuronal class III β-tubulin
