# Supplementary material for: Ferritin nanoparticles for improved self-renewal and differentiation of human neural stem cells
Source: Biomater Res. 2018 Feb 27;22:5. doi: 10.1186/s40824-018-0117-y (PMC5828329; doi:10.1186/s40824-018-0117-y)
Supplement: Supplementary file 1 — Figure S1. The relative viability of hfNSCs in each group after 2 days of culture under self-renewal conditions, which was evaluated by MTT assay (n = 3, *p < 0.05 and **p < 0.01 versus No ferritin group). The viability of each group was normalized to that of the No ferritin group. Figure S2. The relative proliferation of hfNSCs in each group after 2 and 5 days of culture under self-renewal conditions, which was evaluated by MTT assay (n = 3, **p < 0.01 versus No ferritin group). The proliferation of each group at day 5 was normalized to that of each corresponding group at day 2. Figure S3. Immunofluorescence staining of primary hippocampal neurons for Tuj1 (green) and NeuN (red). Cell nuclei were counterstained with DAPI. Scale bar = 200 μm. (DOCX 1587 kb) [file 40824_2018_117_MOESM1_ESM.docx]

**Additional file 1**

**Ferritin Nanoparticles for Improved Self-renewal and Differentiation of Human Neural Stem Cells**

**Authors**:

Jung Seung Lee, Kisuk Yang, Ann-Na Cho, and Seung-Woo Cho^*^

**Affiliations**:

Department of Biotechnology, Yonsei University, 50 Yonsei-ro, Seodaemun-gu, Seoul 03722, Republic of Korea

^*^ Correspondence and requests for materials should be addressed to S.-W.C ([seungwoocho@yonsei.ac.kr](mailto:seungwoocho@yonsei.ac.kr))

**Keywords**

Ferritin, Neural stem cell, Neurosphere, Self-renewal, Differentiation


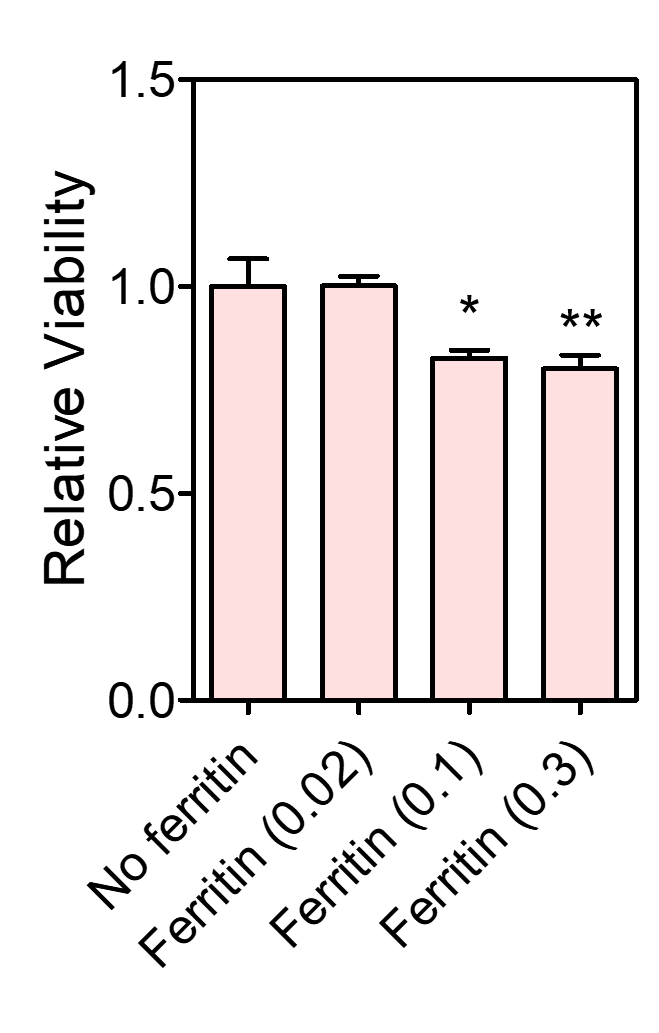


**Figure S1.** The relative viability of hfNSCs in each group after 2 days of culture under self-renewal conditions, which was evaluated by MTT assay (n = 3, **p* < 0.05 and ***p* < 0.01 versus No ferritin group). The viability of each group was normalized to that of the No ferritin group.


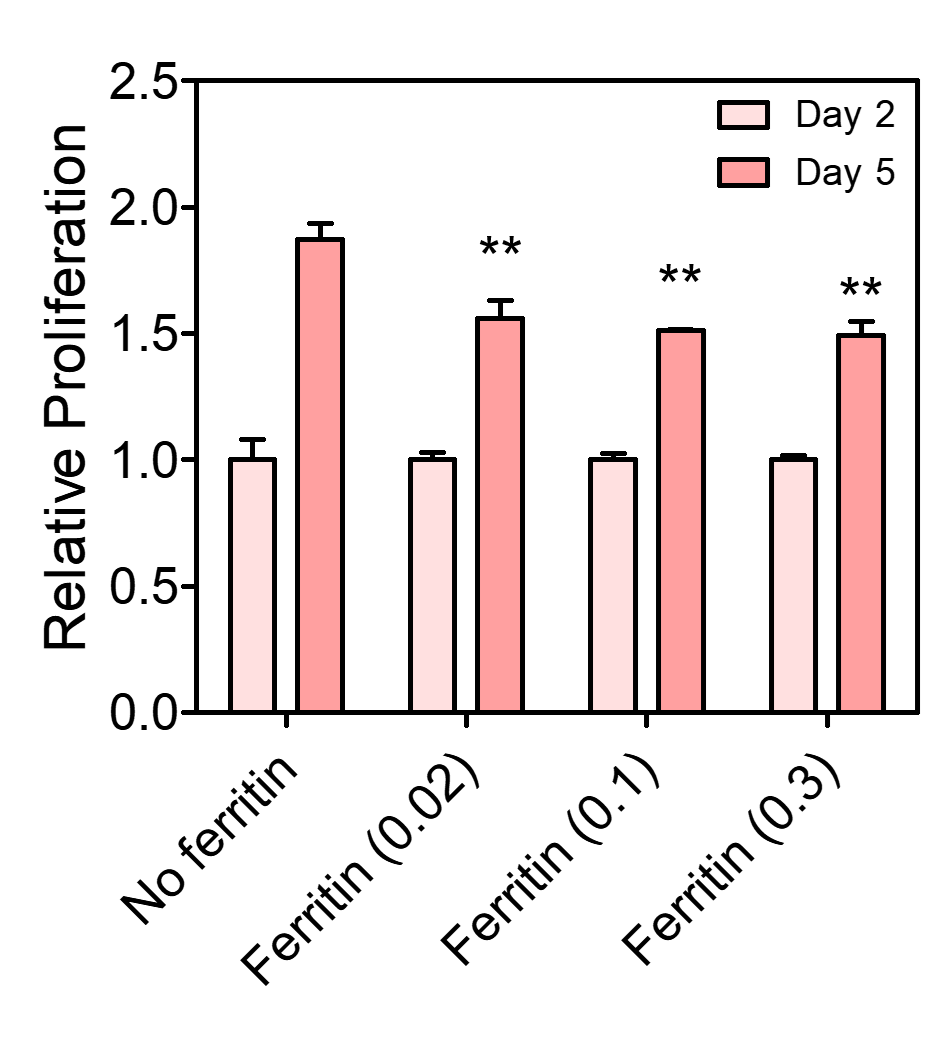


**Figure S2.** The relative proliferation of hfNSCs in each group after 2 and 5 days of culture under self-renewal conditions, which was evaluated by MTT assay (n = 3, ***p* < 0.01 versus No ferritin group). The proliferation of each group at day 5 was normalized to that of each corresponding group at day 2.


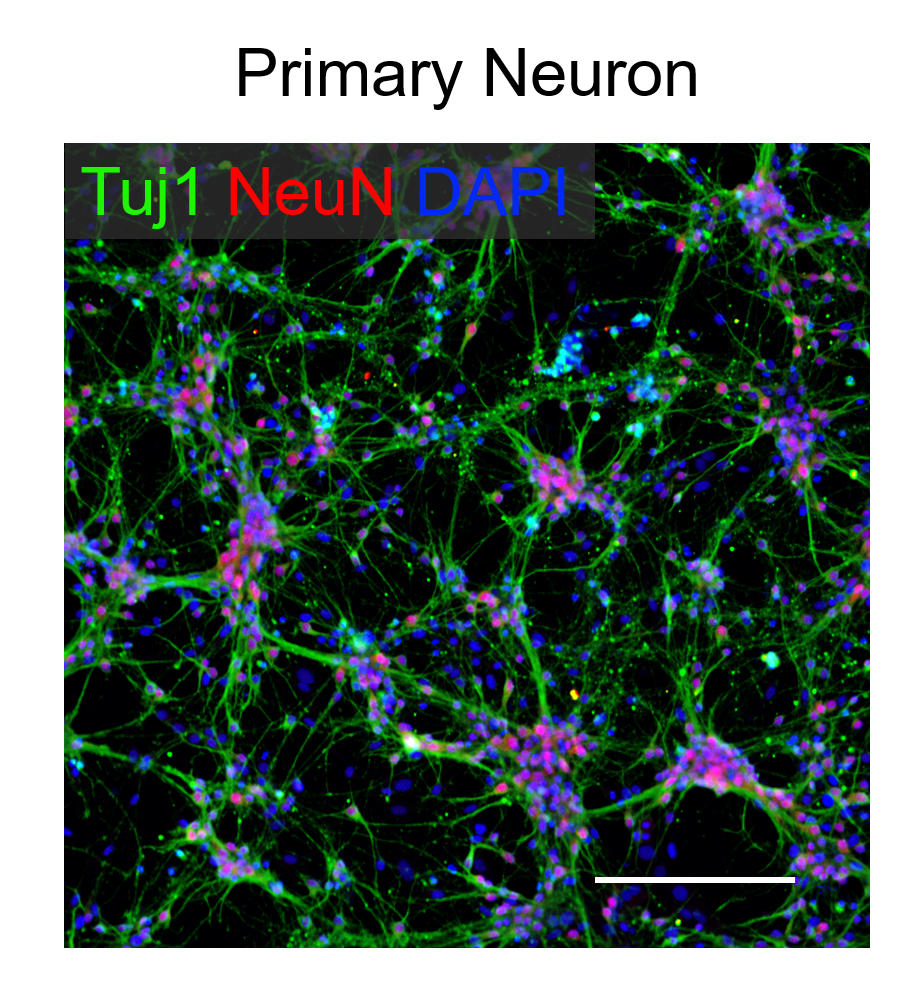


**Figure S3.** Immunofluorescence staining of primary hippocampal neurons for Tuj1 (green) and NeuN (red). Cell nuclei were counterstained with DAPI. Scale bar = 200 μm.
